# Adequacy and complication rates of percutaneous renal biopsy with 18- vs. 16-gauge needles in native kidneys in Chinese individuals

**DOI:** 10.1186/s12882-020-01987-3

**Published:** 2020-08-12

**Authors:** Weiji Xie, Jing Xu, Yi Xie, Zhijun Lin, Xiaochang Xu, Xialan Zhang, Yimin Zhang

**Affiliations:** 1 Department of Nephrology, The Second Affiliated Hospital of Shantou University Medical College, Shantou, Guangdong China; 2 Department of Nephrology, The Sixth Affiliated Hospital of Sun Yat-sen University, Guangzhou, 510655 Guangdong China; 3 Department of Gynaecology and Obstetrics, The Second Affiliated Hospital of Shantou University Medical College, Shantou, 515042 Guangdong China

**Keywords:** Percutaneous, Renal biopsy, Needles, Complication, Adequacy

## Abstract

**Background:**

Percutaneous renal biopsy (PRB) is the primary biopsy technique and it was used by 16G needles or 18G needles in China, but there is controversy about the effect and safety of the two different diameters. The study aims to compare the adequacy, complication rate and pathological classification when using 18G vs. 16G needles to perform renal biopsy with ultrasound-guidedance on native kidneys in Chinese individuals.

**Methods:**

We retrospectively analyzed the number of glomeruli, adequate sample rates, complication rates and pathological classification in 270 patients with the use of 18G or 16G needles from January 2011 to May 2017 and verified whether the needle gauge affected the disease diagnosis.

**Results:**

A total of 270 kidney biopsies were performed. Among them,72 were performed with 18G needles, and 198 were performed with 16G needles. There was no difference in the number of glomeruli under light microscope using 18G relative to 16G needles (24 ± 11 vs. 25 ± 11, *p* = 0.265), whereas more glomeruli were found in the 16G group than in the 16G group using immunofluorescence microscopy (3 ± 2 vs. 5 ± 3, *p* < 0.05). There was no significant difference in the adequate sample rates between the 18G group and the 16G group (90.28% vs. 93.94%, *p* = 0.298). Minor complications including the incidence of lumbar or abdominal pain (4.17% vs. 7.07%, *p* = 0.57), gross hematuria (4.17% vs. 3.54%, *p* = 0.729), and perinephric hematoma without symptoms (4.17% vs. 1.52%, *p* = 0.195), were not significantly different between the 18G and 16G groups. In the 16G group, 2 cases of serious complications occurred: severe gross hematuria requiring blood transfusion and retroperitoneal hematoma requiring surgery. No serious complications were observed in the 18G group, although there was no significant difference in serious complications rates between the 18G and 16G groups (0% vs. 1.02%, *p* = 1).

**Conclusion:**

There was no significant difference in the number of glomeruli, adequate sample rates, or complication rates when using 18G or 16G needles to perform renal biopsy, and the use of an 18G needle with a smaller diameter did not affect the pathological diagnosis or classification of IgA nephropathy and lupus nephritis.

## Background

Percutaneous renal biopsy (PRB) is the primary biopsy technique and was developed by Alwall in 1944 [1]. PRB is an important technique for the diagnosis of kidney disease. The gauge of the needle used for PRB varies from 14G to 22G [2–5]. At present, 16G needles or 18G needles are used for percutaneous renal biopsy in China, but there is still controversy about the effect and safety of 18G and 16G biopsy needles in terms of the two different diameters. Therefore, we retrospectively studied the number of glomeruli, adequate sample rates, complication rates, disease spectrum and pathological classification in patients who underwent renal biopsy with the use of 18G and 16G needles to compare the differences when using these biopsy needles.

## Methods

### Patients-

Patients were undergoing PRB at the Sixth Affiliated Hospital of Sun Yat-sen University, Department of Nephrology, and the Second Affiliated Hospital of Shantou University Medical College, Department of Nephrology from January 2011 to May 2017. The present study was approved by the ethics committee of the Sixth Affiliated Hospital of Sun Yat-sen University and adhered to the tenets of the Declaration of Helsinki.

### Instruments and methods

The Pajunk Delta Cut Biopsy System was applied with the use of 16G and 18G biopsy needles. Ultrasound, routine blood examinations, liver and kidney function tests, and blood coagulation function tests were performed before puncture. Renal biopsy contraindications including platelet count< 120 × 10^3^/uL, low prebiopsy hemoglobin< 12 mg/dL, prothrombin time (PT) extended more than 4 s, acivated partial thromboplastin time (APTT) extended more than 10 s, elevated international normalized ration (INR), and use of anticoagulation including aspirin, warfarin, heparin, and direct factor Xa inhibitors were excluded.

Selection of needle gauge: Prior to February 2015, renal biopsies for patients with relative contraindications (renal insufficiency, coagulopathy, and small kidney size (length ≤ 8 cm)) were performed by using 18G biopsy needles, and biopsies for other patients were performed with 16G needles. Because in the tradition for the transplanted kidney that is precious, so often use 18G biopsy needles. Beside, the experience obtained from renal transplantation biopsy shows that it is possible to completely replace 16G biopsy needle with 18G biopsy needle and the quality of sampling is reliable, but the risk of bleeding will be reduced. So after February 2015, renal biopsies were performed with 18G biopsy needles only. Additionally, we removed the cases in which 18G needles were used due to of relative contraindications. Because of the adjustment of the number of beds in the hospital, the number of biopsies were less performed in the last 2 years of the study.

The biopsies was performed by nephrologists, but ultrasound experts assisted localization. Patients were asked to urinate before PRB. Under the guidance of ultrasound, the lower right renal pole was selected as the biopsy target, and the patient was placed in the prone position. First, disinfection and local anesthesia were performed, then, a guide was placed, and the tip of the needle reached the renal capsule to trigger the biopsy needle and initiate the kidney biopsy. In standard preparation, enough sample must be present to divide into fixative for light, immunofluorescence, and electron microscopy. Usually 2 cores are enough and can be divided into the appropriate fixatives. So this procedure was repeated one more time after drawing. After the operation, the renal biopsy tissue was divided into three sections that were placed under a light microscope, under an electron microscope, and into a fluorescent mirror tube to be preserved for pathological examination. The patient was placed in a supine position 6 ~ 8 h after surgery and was lying in a bed at 24 h. At the same time, the urine color and pain score were recorded and the kidney condition was examined by ultrasonography.

### Observed indicators

① The number of glomeruli was recorded for the 18G and 16G groups, and statistical analyses were performed to evaluate whether the number of glomeruli was significantly different between the two groups. A number of glomeruli ≥10 was set as the standard for an adequate sample, and then a comparison of adequate sample rates was performed between the two groups. Statistical data are presented as the mean ± standard deviation (SD).
② Complications were divided into minor complications and serious complications according to Whittier et al. [6, 7] The minor complications included lumbar or abdominal pain, hematuria, and perirenal hematoma, and serious complications included bleeding requiring surgical intervention or blood transfusion, a decline in blood pressure, a decline in hemoglobin levels, severe infection etc.
③ The pathological diagnosis and pathological classification were compared in the 18G and 16G groups. The Lee classification and Oxford classifications were considered the standards for IgA nephropathy classification. The ISN/RPS2003 classification was considered the standard for lupus nephritis pathological classification [8].

### Statistical analysis

Statistical analysis was performed using IBM SPSS Statistics 22.0 software (IBM Corporation, USA). Measurement data are presented as the mean ± standard deviation, and the comparison was analyzed with the Mann-Whitney U test. The enumeration data were compared with Fisher’s exact test. When the *P*-value was less than 0.05, the difference was statistically significant.

## Results

In this study, a total of 270 patients aged 9 ~ 85 years with an average age of 40 years met the inclusion criteria, there were 152 males and 118 females. In the 18G group, 72 patients with 38 males and 34 females participated, with a mean age of 41 ± 16 years and an average age of 41 years. In the 16G group,198 patients with 114 males and 84 females participated, with a mean age of 40 ± 16 years and an average age of 40 years. There were no significant differences in age, sex or BMI between the two groups. The levels of serum creatinine, hemoglobin, platelets, PT, APTT and urinary protein also did not differ significantly between the two groups before renal biopsy (Table 1).

**Table 1:** Baseline demographic and laboratory features in the 18G and 16G groups

	18G	16G	p
Male	38 (53%)	114 (58%)	
Female	34 (47%)	84 (42%)	0.482
Age	41 ± 16	40 ± 16	0.756
Creatinine (μmol/L)	255 ± 242	216 ± 316	0.11
Hemoglobin(g/L)	116 ± 28	118 ± 26	0.394
Platelets number(*10E9/L)	231 ± 82	248 ± 88	0.084
PT	12 ± 1.8	13 ± 1.2	0.279
APTT	28 ± 5.5	29 ± 4.7	0.466
Proteinuria (g/24 h)	3.3 ± 4.3	3.2 ± 4.2	0.821
BMI (kg/m^2^)	24.73 ± 5.19	23.71 ± 4.29	0.991

*PT* prothrombin time, *APTT* activated partial thromboplastin time

The number of glomeruli was similar between the 18G group and the 16G group as detected by light microscopy (24 ± 11 vs. 25 ± 11, *p* = 0.265), whereas fewer glomeruli were detected in the 18G group than in the 16G group according to the immunofluorescence examination (3 ± 2 vs. 5 ± 3, *p* < 0.05) (Table 2). The adequate sample rates of the 18G group and the 16G group were not significantly different (90.28% vs. 93.94%, *p* = 0.298) (Table 2). Adequate sample rates were not so high in both groups, it may be related to the core length of the sample. The total and immunofluorescence core length of kidney biopsy between the 18G group and the 16G group were not significantly different (2.30 ± 0.58 vs 2.29 ± 0.44, *p* < 0.05; 0.22 ± 0.14 vs 0.25 ± 0.12, *p* < 0.05) (Table 3).

**Table 2:** Adequacy of renal biopsy

	18G	16G	P
LM	24 ± 11	25 ± 11	0.265
IF	3 ± 2	5 ± 3	0.003
LM ≥10	65 (90.28%)	186 (93.94%)	0.298

*LM* Light microscopy glomeruli count, *IF* Immunofluorescence microscopy glomeruli count

**Table 3:** Length of kidney biopsy core

	18G	16G	P
Total core (cm)	2.30 ± 0.58	2.29 ± 0.44	0.411
IF core (cm)	0.22 ± 0.14	0.25 ± 0.12	0.054

*IF* Immunofluorescence microscopy glomeruli

The total complications rate observed in this study was 12.9% (34/270 individuals), and the percentage of complications was similar in the 18G and 16G groups. The incidence of lumbar or abdominal pain in the 18G and 16G groups (4.17% vs. 7.07%, *p* = 0.572), of mild gross hematuria (4.17% vs. 3.03%, *p* = 0.729), and of small hematoma (4.17% vs. 1.52%, *p* = 0.195) were not significantly different. In the 16G group, 2 cases of severe complications occurred. One patients had severe gross hematuria, and blood transfusion was required, the other patient had retroperitoneal hematoma and required renal artery embolization. In the group of patients who underwent the procedure with the use of an 18G needle, there were no serious complications, and the rate of serious complications was 1.02% in the group of patients who underwent the procedure with the use of an 16G needle (0% vs 1.02%, *p* = 1) (Table 4).

**Table 4:** Comparative analysis of biopsy complications N(%)

	18G	16G	p
Minor complications			
lumbar or abdominal pain	3 (4.17%)	14 (7.07%)	0.572
gross hematuria	3 (4.17%)	6 (3.03%)	0.704
perinephric hematoma without symptoms	3 (4.17%)	3 (1.52%)	0.195
serious complications	0 (0%)	2 (1.02%)	1
Total complications	9 (12.5%)	25 (12.63%)	1
Total number	72	198	

Regarding the disease spectrum, the proportion of patients with lupus nephritis in the 18G group was significantly higher than that of patients in the 16G group (16.67% vs. 7.58%, *p* < 0.05). The proportions of other diseases (minor glomerular abnormalities, renal amyloidosis, ischemic renal injury, immunotactoid glomerulopathy, idiopathic nodular mesangial sclerosis, transplant nephropathy, thrombotic microvascular disease due to scleroderma, Alport syndrome, and focal necrotic glomerulonephritis) in the 18G group were lower than those in the 16G group (1.39% vs. 9.6%, *p* < 0.05) (Table 5).

**Table 5:** Comparative analysis of disease spectrum N (%)

	18G	16G	p
IgA nephropathy	25 (34.72%)	62 (31.31%)	0.659
Membranous nephropathy	12 (16.67%)	42 (21.21%)	0.493
Minimal change disease	3 (4.17%)	20 (10.10%)	0.145
Lupus Nephritis	10 (16.67%)	15 (7.58%)	0.038
Diabetic nephropathy	5 (6.94%)	10 (5.05%)	0.554
FSGS	2 (2.78%)	8 (4.04%)	1
Mesangioproliferative glomerulonephritis	1 (1.39%)	6 (3.03%)	0.679
Crescentic glomerulonephritis	4 (5.56%)	3 (1.52%)	0.084
Acute tubulointerstitial injury	2 (2.78%)	7 (3.54%)	1
Hypertension nephropathy	2 (2.78%)	1 (0.51%)	0.174
Chronic sclerosing glomerulonephritis	3 (4.17%)	2 (1.01%)	0.12
Endocapillary proliferative glomerulonephritis	1 (1.39%)	2 (1.01%)	1
Anaphylatic purpura nephritis	1 (1.39%)	1 (0.51%)	0.463
Others^a^	1 (1.39%)	19 (9.6%)	0.019

^a^Others: minor glomerular abnormalities, renal amyloidosis, ischemic renal injury, immunotactoid glomerulopathy, idiopathic nodular mesangial sclerosis, transplant nephropathy, thrombotic microvascular disease due to scleroderma, Alport syndrome, focal necrotic glomerulonephritis

## Discussion

Renal biopsy plays an irreplaceably important role in the diagnosis of kidney disease, the treatment options for kidney disease, and the determination of the prognosis of the disease during renal medicine clinical work. At present, percutaneous renal biopsy is an essential technique for renal medicine, and the safety and success rates of PRB have been further improved after the introduction of automatic biopsy needles and real-time ultrasound guidance [9–11]. Commonly, puncture needles are divided into two categories, namely, negative pressure suction needles and cutting needles. However, the best needle size for the most widely used Tru-Cut needle has not yet been established. In China, the most commonly used needle gauge is 16G (diameter 1.65 mm, length 22 mm or 15 mm), and we generally believe that this size meets the requirements for renal pathological diagnosis. In addition, 18G needles (diameter 1.27 mm, Length 22 mm or 15 mm) have been generally used in patients with relative contraindications (e.g., renal insufficiency, coagulopathy, and small kidney size). Whether an 18G biopsy needle with a smaller diameter is also able to meet the requirements of disease diagnosis and to reduce the incidence of complications remains controversial. The purpose of the retrospective analysis in this study was to clarify this issue.

The premise of accurate pathologic diagnosis is to obtain enough glomeruli such that glomerular numbers can be compared with the use of 18G and 16G needles. It has been reported that 16G needles have a significant advantage over 18G needles in terms of the number of acquired glomeruli [12, 13], while in a prospective study of 14G and 16G biopsy needles, Manno et al [14] found that there was no significant difference in the number of glomeruli obtained in the two biopsies with needles different diameters. We conducted a retrospective analysis and found that under a light microscope, the number of glomeruli was not significantly different between the two groups with the same puncture method described by Manno [14]. In the immunofluorescence examination, the number of glomeruli in the 16G group was slightly higher than that in the 18G group. In general, at least 10 intact glomeruli are required for the assessment of a specimen [15]. Therefore, we set a standard for the samples in order to obtain accurate results; for example, the number of glomeruli had to be ≥10, and adequate sample rates had to be compared between the two groups. We found that the adequate sample rates in the 18G and 16G groups were not significantly different. Thus, there was no obvious difference between the two groups in terms of the number of glomeruli. The core length of the kidney tissue is usually between 1.5-cm and 2-cm. Table 3 excludes the abnormal number of glomeruli caused by sample length, indicating that the difference in the number of glomeruli in the two groups in the immunofluorescence is caused by the width of the sample, and the results are more reliable.

The pathological diagnosis of renal disease depends on the quality of the acquired specimen. When the number of glomeruli in pathological specimens is insufficient, it may affect the diagnosis of renal disease or the classification of some diseases, resulting in misdiagnosis. For example, FSGS is characterized by a focal segmental distribution and is easily missed in the presence of few glomeruli. Therefore, to diagnose a focal disease, more than 20 glomeruli may be needed [7]. However, membranous nephropathy requires only one glomerulus for diagnosis [12]. Generally, biopsies with 16G needles for renal pathological diagnosis can meet the requirements. In the current study, using the distribution of pathological diagnoses in the 16G group as a standard, we performed a comparative analysis of the proportion of pathological diagnoses with 18G and 16G needles to assess the impact of the two different types of needles on the pathological diagnosis and pathological classification. We statistically analyzed the disease spectrum between the two groups and found that the incidence of lupus nephritis was 16.67% in the 18G group, which was much higher than that in the 16G group (7.58%). IgAN, MN, MCD, lupus nephritis, diabetic nephropathy, FSGS, MsPGN, crescentic glomerulonephritis, acute tubulointerstitial injury, hypertension nephropathy, chronic sclerosing glomerulonephritis, endocapillary proliferative glomerulonephritis, and anaphylactic purpura nephritis were found in equal proportions in the two groups, whereas the proportion of other rare diseases was smaller in the 18G group than in the 16G group (Table 5).

The most frequent biopsy finding in China is IgA nephropathy, accounting for 36.3% of all primary glomerulopathies [16]. However, the number of glomeruli may affect the classification of IgA nephropathy, including the Lee and Oxford classifications. Therefore, the difference in the proportion of different classifications of IgA nephropathy is most likely related to the small sample size, which may be related to the size of the needle. To further analyze the influence of needle size on the diagnosis of pathological classification, we comparatively analyzed the pathological classification in the 18G and 16G groups (Tables 6, 7). The proportion of IgA nephropathy with Lee grade IV was higher in the 18G group than in the 16G group (48.00% vs. 24.19%, *p* < 0.05), whereas there was no significant difference in the proportion of other IgA nephropathy pathological classifications between the two groups. Similarly, we chose the new Oxford classification (MEST-C), which was published in 2017 and was added to the cellular/fibrocellular crescent score, and compared it with the older Oxford classification (MEST), which was published in 2009. The results revealed that the proportion of Oxford classifications (MEST-C) of IgA nephropathy with segmental glomerulosclerosis was higher in the 18G group than in the 16G group (52% vs. 24.59%, *P* < 0.05), whereas there was no significant difference in the proportion of other IgA nephropathy pathological indices between the two groups. Furthermore, we evaluated the size of the needle to determine the effect of lupus nephritis classification, and the results showed that there was no significant difference in the ratio of lupus nephritis classification between the two groups (Tables 8). Considering the glomerular count and adequate sample rates of the 18G group and the 16G group, we suggest that a change in needle gauge is not a major cause of change in the proportion of IgA nephropathy in the two groups. In conclusion, through statistical analysis, our results strongly suggest that 18G needles with smaller diameters have no significant impact on the diagnosis and pathological classification of renal diseases.

**Table 6:** Comparative analysis of IgAN classification (SMK Lee classifications) N (%)

	18G	16G	p
Grade I	0	0	
Grade II	0	7 (11.29%)	0.185
Grade III	8 (32%)	26 (41.94%)	0.471
Grade IV	12 (48%)	15 (24.19%)	0.041
Grade V	5 (20%)	14 (22.58%)	1

**Table 7:** Comparative analysis of IgAN classification (Oxford classification^a^, 2017) N (%)

	18G	16G	p
M1	25 (100%)	61 (100%)	1
E1	7 (28%)	25 (40.98%)	0.329
S1	13 (52%)	15 (24.59%)	0.022
T1	7 (28%)	9 (14.75%)	0.221
T2	4 (16%)	12 (19.67%)	0.77
C1	4 (16%)	8 (13.11%)	0.739
C2	3 (12%)	10 (16.39%)	0.748

^a^The Oxford classification consisted of mesangial hypercellularity (M0, mesangial score ≤ 0.5; M1, mesangial score > 0.5), endocapillary hypercellularity (E0, absent; E1, present), segmental glomerulosclerosis (S0, absent; S1, present), tubular atrophy/interstitial fibrosis (T0, ≤25%; T1, 26–50%; T2, > 50%), and cellular/fibrocellular crescents (C0, absent; C1, < 25%; C2, ≥25%)

**Table 8:** Comparative analysis of lupus nephritis classification (the ISN/RPS classification, 2003) N (%)

	18G	16G	p
Class I	0 (0%)	0 (0%)	
Class II	0 (0%)	3 (20%)	0.25
Class III	0 (0%)	0 (0%)	
Class IV	2 (20%)	3 (20%)	1
Class V	0 (0%)	2 (13.33%)	0.5
Class VI	0 (0%)	0 (0%)	
Class III + V	3 (30%)	1 (6.67%)	0.267
Class IV + V	5 (50%)	6 (40%)	0.697

Complications of renal needle biopsy include gross hematuria, lumbar abdominal pain, perirenal hematoma, arteriovenous fistula, infection, etc. [17], with bleeding being the most common complication [18]. Intervention is not required for most of these complications, but severe complications requiring surgical intervention are rare. The incidence of serious complications is approximately 1 to 7% [6, 14, 19, 20], and 0.1% of patients die due to renal biopsy [21]. A question that needs to be addressed in the field is whether it is possible to use a needle with a smaller diameter for PRB to reduce postoperative complications. The correlation between needle diameter and the incidence of postoperative complications remains controversial. Corapi et al [22] found that the risk of bleeding due to large-diameter puncture needles was higher than that due to small-diameter puncture needles, and the bleeding was defined as percent drop in hemoglobin from pre to post procedure. Peters et al [13] concluded that the 16G needle was superior to the 18G needle in terms of the quality of sampling and postoperative complications. Roth and Mai J et al. [18, 23] pointed out that the incidence of postoperative complications was similar in the 16G and 18G groups. In our study, abdominal pain, gross hematuria, and perirenal hematoma were common complications of renal biopsy. Notably, there were two cases of severe complications in the 16G group. The percent drop in hemoglobin in these two cases of severe complications was 25 and 28%. Because complications were relatively rare, the results of mild complications and the incidence of serious complications did not show a statistically significant difference between the two groups.

Because the sample size is not large enough, the pathological diagnosis of the samples obtained in this study just coincides with the clinical diagnosis. In the next study, we can expand the sample size to explore the proper diagnosis rate of renal biopsy for renal disease.

## Conclusions

Taken together, the results show that a 16G biopsy needle has no obvious advantage over an 18G needle in terms of the number of glomeruli and the adequate sample rate, and an 18G needle with a smaller diameter does not affect disease diagnosis. Therefore, an 18G needle has the same application value as a 16G needle and further studies using a larger sample would be useful to confirm these findings.

## Data Availability

The data that support the findings of this study are available from the Sixth Affiliated Hospital of Sun Yat-sen University and the Second Affiliated Hospital of Shantou University Medical College but restrictions apply to the availability of these data, which were used under license for the current study, and so are not publicly available. Data are however available from the authors upon reasonable request and with permission of the Sixth Affiliated Hospital of Sun Yat-sen University and the Second Affiliated Hospital of Shantou University Medical College.

## Abbreviations

PRB: Percutaneous renal biopsy
IgAN: IgA nephropathy
MN: Membranous nephropathy
MCD: Minimal change disease
FSGS: Focal segmental glomerulosclerosis
MsPGN: Mesangioproliferative glomerulonephritis
